# Contingent self-esteem and vulnerability to depression: academic contingent self-esteem predicts depressive symptoms in students

**DOI:** 10.3389/fpsyg.2015.01573

**Published:** 2015-10-20

**Authors:** Claudia Schöne, Sarah S. Tandler, Joachim Stiensmeier-Pelster

**Affiliations:** Educational Psychology, Department of Psychology, University of GiessenGiessen, Germany

**Keywords:** contingent self-esteem, depression, adolescence, self-esteem level, gender differences, academic contingency of self-worth, self-esteem, contingencies of self-worth

## Abstract

Low self-esteem has been established as a vulnerability factor for depression. In line with recent research, we suggest that a full understanding of the role of self-esteem in depression requires consideration of contingent self-esteem as well. For most people, competence is an important source of self-esteem. Students in particular link their self-esteem to academic competence. To test the hypothesis that academic contingent self-esteem (aCSE) predicts depressive symptoms (DS), two studies were conducted. Preceding the investigation of our hypothesis, the first purpose of Study 1 was to describe the development of aCSE, self-esteem (SE) level, and DS in adolescence in a sample of German students aged 10–16 (*N* = 1888) in order to provide a foundation for further analyses. Then, to address the main question, age and gender differences in aCSE, SE level, and DS as well as their relations were investigated. The results show that (1) gender differences emerged after the age of 10/11. Girls scored higher on aCSE and DS and lower on SE level than did boys, and aCSE and DS decreased and SE level increased over time in boys, while the rather disadvantageous pattern in girls remained stable. (2) After controlling for SE level and aCSE, the effects of gender and age × gender interaction on DS disappeared, suggesting an influence of aCSE on DS. (3) aCSE predicted DS over and above SE level. Since the results of Study 1 did not allow for causal conclusions, a longitudinal study (*N* = 160) was conducted to further investigate the causal role of aCSE. According to the diathesis-stress model, aCSE was expected to serve as a diathesis for developing DS in the face of academic stress (daily hassles) during an academic semester at university. The results of Study 2 revealed that aCSE interacted with corresponding hassles to predict increases in DS. High levels of academic stress led to increases in DS only among students who strongly based their SE on academic competence. Implications for prevention and intervention of depression are discussed.

## Introduction

Depression is one of the most frequent mental health problems in children and adolescents. Prevalence estimates of depression show an increase in the rate from childhood to adolescence (Essau et al., 2000; Costello et al., 2011). Approximately 11% of children aged between 12 and 13 years have experienced a major depressive episode. Among adolescents aged 16–17 years, almost 25% reported having had a major depressive episode at some time in their lives (Essau et al., 2000). Furthermore, there are consistent findings that incidence rates of depression are nearly twice as high in females as in their male counterparts during adolescence (Essau et al., 2000, 2010; Ihle et al., 2005).

Early onset of depression influences children’s development and is accompanied by impairment in many domains of functioning. Lowered school performance along with poor concentration, lowered thinking ability, fatigue, and psychomotor retardation are within the wide range of potential consequences of depressive symptoms (DS). Furthermore, depressive children and adolescents often experience problems and conflicts with parents, peers, and teachers due to their symptoms (Groen, 2002). Previous studies have shown high psychiatric comorbidities of depression with anxiety, somatoform disorders, ADHD, eating disorders, PTSD, or substance abuse among adolescents (Lewinsohn et al., 1998; Hoffmann et al., 2012). Early age at onset of depression, especially in combination with comorbid disorders, is associated with an elevated risk of the persistence of depression (Groen and Petermann, 2005) as well as a number of negative outcomes in adulthood such as lower life satisfaction, lower educational aspirations, and early marriage (Gotlib et al., 1998; Franko et al., 2005). In sum, these results highlight that many children and adolescents suffer from depression and its consequences. Finding risk factors that play a central role in the etiology of depression is crucial for the development of prevention and treatment programs for children and adolescents with depressive disorders. However, above all, the increasing rates of depression in children and younger adolescents raise important questions about the development of depressive disorders and underscore the importance of identifying vulnerabilities to this disorder at a young age.

In this article, we propose that contingent self-esteem (SE) is related to DS, specifically that this facet of SE predisposes children and adolescents to DS. Drawing on evidence from diathesis-stress models, we suggest that contingent SE represents vulnerability for depression. In the present studies, we focus on children and adolescents in order to investigate the developmental stages in which gender differences in depression emerge. Since very little is known about contingent SE in this age span, providing such information is another preliminary goal of this article.

### Multidimensional Self-esteem and Vulnerability for Depression

A large number of prominent theories in the field of depression have focused on cognitive vulnerability (e.g., Beck, 1983; Abramson et al., 1989). One of the most important cognitive variables relating to depression is SE. Within vulnerability models, low SE level is assumed to predispose people to DS (Beck et al., 1986; Brown and Harris, 2001). In support of these vulnerability models, previous research has provided evidence that low SE predicts subsequent levels of DS in adolescence and young adulthood (Orth et al., 2008, 2009). Critics argue, however, that low SE is a key symptom of major depression; thus, it is questionable whether low SE is a symptom of or a vulnerability factor for depression (Burwell and Shirk, 2006).

In recent years, a growing number of studies have adopted a more multidimensional concept of SE. Specifically, some investigators have focused on stability of SE and contingent SE as other important aspects of SE (Deci and Ryan, 1995; Crocker and Wolfe, 2001; Kernis, 2003). By following the multidimensional approach of SE, previous studies have found that stability and contingent SE are important predictors of DS (Kernis et al., 1998; Burwell and Shirk, 2006; Franck and De Raedt, 2007). In the present paper, we focus on contingent SE and its relation to depression in early and late adolescence.

### Contingent Self-esteem in Adolescence

According to Deci and Ryan (1995, p. 32) “contingent self-esteem refers to feelings about oneself that result from – indeed, are dependent on – matching some standard of excellence or living up to some interpersonal or intrapsychic expectations.” Thus, SE varies on a dimension from non-contingent to strongly contingent (cf. Kernis, 2003). Crocker et al. (2003b) and Crocker and Park (2004) focus on specific contingencies on which a person’s SE is based, such as others’ approval or academic competence. This approach focuses on individual differences in the domain that people perceive as relevant for their SE—in other words, the domains they have to master to be a worthy person. For individuals with high contingent SE, success or positive events within that domain lead to an increases in state SE, whereas failures lead to a drop (Crocker et al., 2002, 2003a). Thus, people with contingent SE have rather fragile or vulnerable SE (Crocker and Park, 2004; Sanchez and Crocker, 2005). Moreover, SE that requires validation—especially when such validation is beyond one’s control—is easily threatened and leads to maladaptive pursuit of SE (Park and Crocker, 2005). The pursuit of SE in turn generates further fragile SE and undermines mental and physical health. In line with this argument, preliminary evidence suggests that contingent SE is associated with negative affect (Vonk and Smit, 2012), lower levels of SE, higher rates of neuroticism (Crocker and Luhtanen, 2003; Maricutoiu et al., 2012), and mental and physical health problems (DiBartolo et al., 2004; Neighbors et al., 2004; Sanchez and Crocker, 2005). In sum, contingent SE must be considered to fully understand the link between SE and depression.

With regard to the development and reorganization of contingent SE, adolescence might prove to be a critical stage because of the changes in awareness of one’s self and others’ reactions to it (Burwell and Shirk, 2006). Furthermore, adolescents are faced with many developmental tasks and challenges, such as creating a stable identity, acquiring a set of values, disturbances in relationships, and increased performance demands and evaluative pressure. These challenges in combination with existing and evolving contingent SE in various domains might be a main reason for the increase in DS during the transition period from childhood to adolescence. Thus, assessing contingent SE at different developmental stages is essential for a better understanding of the process underlying the onset of depressive disorders.

Typically, depression increases during puberty; however, this increase seems to be greater among girls, which leads to gender differences in depression. With contingent SE being an important factor in the development of depression, it is crucial to obtain knowledge about the development of DS and especially about the development of SE level and contingent SE in adolescents (both pre-pubescent and post-pubescent). With regard to level of SE in adolescents, research has yielded surprisingly inconsistent results (see also Robins et al., 2002; Erol and Orth, 2011). A longitudinal study reported a cubic effect of time on SE level for both male and female students: SE among females increased until age 12, after which it decreased until age 17, while SE level among males increased until age 14, decreased until about age 16, and then increased again after age 16 (Baldwin and Hoffmann, 2002). Since the onset of puberty occurs slightly earlier for girls than for boys, the overall picture could be interpreted as follows: SE increases until the onset of puberty and decreases thereafter, paralleling the known age trajectory of depression. Considering large-scale studies from the last 15 years, research findings indicate that SE increases during adolescence (from the age of 14; Erol and Orth, 2011) or stays roughly the same between the ages of 14 and 18 years (Bachman et al., 2011).

While the results for SE level are mixed, very little is known about age-group differences and the trajectory of contingent SE in adolescence. Mainly, there is a lack of research on contingent SE in adolescence, especially in early/mid adolescence. In addition, in most of the very few studies where contingent SE actually has been investigated in samples of adolescents, only sample means were reported, since age differences were outside the scope of these studies (e.g., Bos et al., 2010; Wouters et al., 2013a). To our knowledge, there are only two studies in which contingent SE was investigated and where scores for age-related subsamples were reported. Burwell and Shirk (2006) found contingent SE to be temporally highly stable over a period of half a year among young adolescents aged 12–15 years. In a second study by Meier et al. (2011) wherein contingent SE was operationalized in a rather indirect way as “the degree to which an individual’s daily self-esteem and affect fluctuates in response to conflicts occurring on the same day”, contingent SE was found to decrease from 7th to 10th grade. However, these results should be interpreted with some caution, as they do not directly represent contingent SE.

In sum, there is not much knowledge about the development of contingent SE. Therefore, a preliminary aim of this study is to provide information by investigating contingent SE (as well as SE level) among adolescents.

### Contingent Self-esteem and Depression in Adolescence

Although some theories have linked contingent SE to depression (Crocker and Wolfe, 2001; Crocker and Park, 2004; Cambron et al., 2009), empirical evidence about the causal relation of DS and self-worth contingencies is comparatively limited. Furthermore, previous research primarily focused on college students aged 16 and older. Thus, there is a lack of knowledge about the development of contingent SE among children and young adolescents (as depicted above) and its relation to DS. The results of two studies with college samples aged 17–22 years (Crocker et al., 2002, 2003a) demonstrated that students with higher academic contingent self-esteem (aCSE) experienced greater fluctuations in state SE in response to academic success and failure than did students with lower aCSE. Instability of SE, in turn, was a predictor of DS for students who were initially more depressed (Crocker et al., 2003a). Some studies have provided further and more direct evidence for the vulnerability hypothesis of contingent SE. Specifically, contingent SE was a positive predictor of DS in college students (Cambron et al., 2010) and adults (Soenens and Duriez, 2012). Whereas these studies used cross-sectional data, other researchers investigated the relation between contingent SE and increases in DS with a longitudinal design. Their findings indicated that external contingent SE at the start of an academic semester predicted DS at the end of the semester after controlling for initial DS (Sargent et al., 2006; Lopez et al., 2014). In conclusion, empirical research using adult and late adolescent samples has provided promising initial results supporting the hypothesis that contingent SE is a vulnerability factor for depression.

However, most of these studies did not investigate unique predictive (i.e., incremental) effects of contingent SE beyond mere SE level (Bos et al., 2010; Wouters et al., 2013b; Sowislo et al., 2014). Because of the close conceptual relation between contingent SE and SE level as well as the high association between SE level and depression, it seems to be possible that the effects of contingent SE on depression are explained by SE level (in other words, the association of contingent SE with SE level). Also, the effects of SE on depression might be in part due to contingent SE. Therefore, a necessary step is to test the predictive value of contingent SE on depression beyond mere SE level.

In cognitive diathesis-stress models, vulnerability is an internal feature of a person that predisposes the individual to DS following the occurrence of negative life-events. In other words, when the vulnerable individual is confronted with negative events or challenging life conditions, DS are likely to emerge (cf. Abela and Hankin, 2008). Thus, it is the interaction between a person’s vulnerability and negative events that produces DS. According to the assumptions of diathesis-stress models, adolescents with contingent SE are vulnerable to the development of DS, when they experience negative events congruent with the domain of their contingency. However, to date, little is known whether contingent SE interacts with corresponding stressors in predicting DS. Cambron and Acitelli (2010), for example, investigated the interaction effects of contingent SE and negative events on DS. Contrary to the assumption of diathesis-stress models, the interaction failed to predict DS in a student sample using cross-sectional data. According to our knowledge, there is only one longitudinal study that examined a diathesis-stress model of contingent SE as a vulnerability to depression among a sample of adolescents (Burwell and Shirk, 2006). This study revealed that the interaction between social contingent SE and social stress has a significant positive effect on increases of DS. However, there was no significant interaction between aCSE and academic stress. In sum, previous research on diathesis-stress models of contingent SE is rare and has revealed only mixed results thus far.

However, there are some promising findings supporting the hypothesis that contingent SE predisposes children and adolescents to develop DS. Nonetheless, some research questions remain unanswered. First, it is unclear whether increases in depression during puberty (which occurs most often among girls) are caused by an increase in contingent SE. If contingent SE explains those differences, this could indicate a causal relation. Second, there are only mixed results in support of a diathesis-stress model of contingent SE. Moreover, the incremental value of contingent SE compared to SE level is uncertain. Finally, with respect to adolescents, there is a need for information about the development of contingent SE in this age span.

## Study 1

### Overview

In this self-report-questionnaire-based cross-sectional study, we investigate the relation between DS and contingent SE in adolescents. Due to the known increase in DS in adolescence, we consider this developmental stage, especially the transition to puberty, useful for the investigation of causal factors of depression such as contingent SE. Based on the reasons outlined above, we hypothesize that the development of DS, including the emerging gender differences, is in part due to contingent SE. Adolescents (and people in general) who base their SE on meeting standards are presumably more vulnerable to developing DS compared to those whose SE is mostly unconditional and “true”. While we consider our reasoning valid for contingent SE in general, we chose to test our assumptions for the domain of academic competence. As outlined above, aCSE has been shown to be a predictor of DS in college students, but not yet in adolescents. Among other domains, academic competence and performance (i.e., doing well in school and demonstrating competence) likely plays an important role in adolescents’ lives. In addition to considering oneself to be physically attractive and popular in school, considering oneself to be smart and competent is in general an important source of SE. Another reason why we chose the academic domain is that adolescents spend a significant amount of time at school, where negative events (stressors) are ubiquitous (e.g., not mastering a task, negative performance feedback from teachers, direct and indirect comments on their abilities, or giving an incorrect answer in class); in view of the underlying theoretical model, this is an important presumption.

Study 1 serves two purposes. Before investigation of our main hypothesis, we explored the development of aCSE, SE level, and DS in a sample of adolescents aged 10–16, with the students aged 10 and 11 representing prepubescents. In doing so, we intended to obtain knowledge specifically on contingent SE in adolescents in order to provide a background for further analyses. Since very little is known about contingent SE in adolescence, it is rather difficult to generate precise expectations concerning the development. The trajectory of DS in adolescence is clearer: typically, depression increases during puberty, especially among girls. We expect to find an increase in both contingent SE and DS during puberty, accompanied by a decrease in SE level (especially among girls). Gender differences are expected to emerge with the onset of puberty.

The second purpose of Study 1 was to contribute to the investigation of our main hypothesis that contingent SE fosters the development of DS. We further analyze this in terms of gender × age interactions. We assume that the typical gender and age differences in depression (specifically, the increase in girls and after the onset of puberty) are due to contingent SE. If this assumption proves correct, gender and age effects would be reduced when controlling for contingent SE and SE level. At the same time, contingent SE is supposed to have a positive effect on DS. This pattern would provide preliminary support for our hypothesis that contingent SE is a risk factor for DS. We additionally controlled for SE level in order to examine whether contingent SE predicts DS over and above the SE level.

### Method

#### Participants and Procedure

The sample comprised *N* = 1888 students (942 female) from 26 secondary schools spread across Germany. Students aged 10–16 years (*M* = 13.44, *SD* = 1.76) completed questionnaires in class with their teachers absent. All instructions and questionnaires were administered in German by trained graduate-level psychology students. Permission to conduct our study was given by the ministries of education and research, from all participating schools, and from the students’ parents. In addition, before the assessment, students were informed that participation was voluntary. Students were not compensated for participating.

#### Measures

##### Academic contingent self-esteem and *self-esteem* level

Academic contingent self-esteem and SE level were measured with two scales of the *Selbstwertinventar für Kinder und Jugendliche – SEKJ* (Schöne and Stiensmeier-Pelster, in press). The SEKJ is a recently developed German SE inventory for children and adolescents. It was statistically validated and standardized in a sample of 3100 German children and adolescents aged 10–17. The construct validity was substantiated by the pattern of factor loadings and covariations with stability of SE, trait test anxiety, rumination, ability self-concept, DS, and aCSE or SE level respectively (Schöne and Stiensmeier-Pelster, in press). Participants first completed the 10-item self-report measure of SE. This scale assesses SE level as global self-worth with a focus on emotional positive self-regard (“*I like myself*”) and affective evaluation of the self. This scale is similar to the Rosenberg Self-Esteem Scale (RSES; Rosenberg, 1965), except it does not contain the RSES’s references to competence and performance (e.g., “*I am able to do things as well as other people*”). The participants then completed a 12-item measure of aCSE. The items included both performance and competence as contingencies of SE. Like established questionnaires for college students (Crocker et al., 2003b), the items measure aCSE through self-reported increases or decreases in state SE following positive/negative events regarding competence (“*When I consider myself incompetent, I feel worthless*”) or performance (“*When I get better grades at school than my classmates, I somehow feel more worthy*”) at school. All items were rated on a scale ranging from 1 (*strongly disagree*) to 5 (*strongly agree*). High values indicate high SE and high aCSE. The Cronbach’s alphas were as follows: α_SE_ = 0.87 and α_aCSE_ = 0.86.

##### Depressive symptoms

Depressive symptoms were measured with the *Depressionsinventar für Kinder und Jugendliche* (DIKJ; Stiensmeier-Pelster et al., 2014), an established German self-report inventory for measuring the severity of DS in children and adolescents aged 8–16 years. Conceptually, the DIKJ is originally based on the Children’s Depression Inventory (CDI; Kovacs, 1985). The items cover all relevant symptoms of depression mentioned in the DSM-5 (American Psychiatric Association [Apa], 2013) such as depressed mood (sadness, hopelessness, and irritability), loss of interest or pleasure, fatigue or loss of energy, diminished ability to concentrate, loss of appetite, and social withdrawal, but not suicide ideation or thoughts about death. Only one item out of 29 seemed related to SE (“*I hate myself*”). Other example items are “*I seldom feel sad or miserable* [0],” “*I often feel sad or miserable* [1],” and “*I always feel sad or miserable* [2]” and “*I take much pleasure in many things* [0],” “*I only take pleasure in a few things* [1],” and “*I don’t take pleasure in anything* [2].” Responses were measured on a 3-point scale (0 = no symptoms at all, 1 = shows some symptoms, 2 = shows pronounced symptoms) and, after recoding, ratings are summed to produce a total score. The possible range of scores is 0–58, with higher scores indicating greater DS. According to the test manual, the recommended cut-value is 20. The internal consistency for the 29-item scale was Cronbach’s α = 0.88.

##### Age groups

Since we were interested in the developmental changes brought on by puberty, the sample was split into three age groups: a prepubescent group (aged 10;1–11;11 years; *n* = 338), and two groups with an age range from 12;1–13;11 years (*n* = 558) and age 14;1–16;11 years (*n* = 990). Without biological markers, it is rather impossible to determinate and mark the individual time of puberty correctly. Based on data from the German KiGGS study (a large-scale, long-term study on the health of children and adolescents in Germany), we concluded that age 12–13 best represents the phase of onset of puberty. According to the KiGGS study, the mean age in girls for menarche is 12 years 8 months, and the median age in boys for voice mutation is 13 years 5 months (Kahl et al., 2007). Thus, this age group likely incorporated the average age in which German girls and boys experience the most significant developmental changes linked to puberty.

### Results and Discussion

#### Descriptive Statistics and Intercorrelations

The mean scores and standard deviations of SE level, aCSE, and DS as well as scale intercorrelations are presented in **Table 1**. The mean scores for SE level were above the theoretical scale mean of 3, while the mean scores for aCSE were slightly below the theoretical scale mean. For both variables, this is similar to the values reported in the test manual (Schöne and Stiensmeier-Pelster, in press). Reported means of DS indicate that—after applying the recommended cut-off-score (>20) (Stiensmeier-Pelster et al., 2014)—17.4% of the sample were considered depressed.

**Table 1:** Possible range, mean scores, and intercorrelations of self-esteem (SE) level, academic contingent self-esteem (aCSE), and depressive symptoms (DS).

	Range	Mean	1	2	3
(1) Self-esteem level	1–5	3.73	—		
(2) Academic contingent self-esteem	1–5	2.70	-0.37^∗∗^	—	
(3) Depressive symptoms	0–56	14.09	-0.63^∗∗^	0.37^∗∗^	—

*^∗∗^*p* < 0.001.*

There was a rather large negative correlation between DS and SE level, and a moderate positive correlation between DS and aCSE. Correlations between SE level and aCSE were moderate and negative (see **Table 1**).

#### Primary Analyses

First, aCSE, SE level, and DS were described and examined by age and gender (see **Table 2**). Three analyses of variance (ANOVA) on DS, SE level, and aCSE were conducted with the factors of gender (male vs. female) and age (age 10–11 vs. age 12–13 vs. age 14–16).

**Table 2:** Mean scores and standard deviations of self-esteem (SE) level, aCSE, and DS by age and gender.

	Age 10–11 (*n* = 338)		Age 12–13 (*n* = 558)		Age 14–16 (*n* = 990)		
	Male	Female	Male	Female	Male	Female	Total sample
	*n* = 175	*n* = 163	*n* = 283	*n* = 275	*n* = 486	*n* = 504	(*n* = 1888)
	*M (SD)*	*M (SD)*	*M (SD)*	*M (SD)*	*M (SD)*	*M (SD)*	*M (SD)*
SE level	3.97 (0.73)	3.76^a,b^ (0.81)	3.83 (0.74)	3.56^a^ (0.81)	3.92 (0.74)	3.50^b^ (0.79)	3.73 (0.79)
aCSE	2.65 (0.72)	2.75 (0.84)	2.68^a^ (0.75)	2.84 (0.76)	2.50^a^ (0.74)	2.84 (0.81)	2.70 (0.78)
DS^1^	11.69^a^ (7.64)	12.45^b,c^ (7.20)	14.31^a,d^ (7.83)	16.73^b^ (9.37)	12.26^d^ (7.39)	15.69^c^ (7.86)	14.40 (8.10)

*Same letters within rows indicate statistically significant differences at *p* < 0.05; ^1^possible range: 0–58, *Md* = 12.5, cut-off value: 20.*

##### Self-esteem level

The two-way ANOVA on SE level revealed statistically significant main effects for gender (*F*[1,1880] = 59.05, *p* < 0.001, η^2^ = 0.03) and age (*F*[2,1880] = 6.10, *p* = 0.002, η^2^ = 0.01), as well as a statistically significant age × gender interaction (*F*[2,1880] = 3.58, *p* = .03, η^2^ = 0.004). To further investigate the age × gender interaction, two one-way ANOVAs with the three age groups as factors were conducted separately for males and females. The analyses yielded an age group difference for females (*F*[2,939] = 6.41, *p* = 0.002, η^2^ = 0.01), but not for males (*F*[2,940] = 2.45, *p* = 0.087, η^2^ = 0.005). *Post hoc* tests using Scheffé’s method revealed that—for female students—SE level decreased from age 10–11 to age 12–13 (*p* = 0.002), and then remained stable at this lower level from age 12–13 to age 14–16 (*p* = 0.656). See **Table 2** for means and standard deviations. Male students reported higher SE than did female students (*M* = 3.56, *SD* = 0.80 vs. *M* = 3.90, *SD* = 0.74) in all three age groups (all *p* < 0.01). The gender differences increased with age from very small to medium (Cohen’s *d_1_* = 0.10, *d_2_* = 0.28, *d_3_* = 0.45).

##### Academic contingencies of self-worth

The two-way ANOVA on aCSE yielded a statistically significant main effect for gender (*F*[1,1880] = 25.98, *p* < 0.001, η^2^ = 0.02), but not age (*F*[2,1880] = 2.655, *p* = 0.071, η^2^ = 0.003). As expected, the results revealed an age × gender interaction on aCSE (*F*[2,1880] = 4.30, *p* = 0.014, η^2^ = 0.005). To further investigate the interaction, two one-way ANOVAs (factor: three age groups) were conducted separately for males and females. These analyses showed a statistically significant age group difference in males (*F*[2,941] = 6.54, *p* = 0.002, η^2^ = 0.014), but, unexpectedly, not in females (*F*[2,939] = 0.86, *p* = 0.423, η^2^ = 0.002). See **Table 2** for means and standard deviations. *Post hoc* tests using Scheffé’s method showed that, in male students, aCSE did not change from the age 10–11 to age 12–13 (*p* = 0.926), but decreased slightly from age 12–13 to age 14–16 (*p* = 0.004); however, the difference between age groups 10–11 vs. 14–16 was not statistically significant (*p* = 0.058). Male students reported lower aCSE than did female students at age 12–13 (*t*[556] = -2.53, *p* = 0.012) and age 14–16 (*t*[556] = -6.87, *p* < 0.001), but not at age 10–11 (*t*[336] = -1.13, *p* = 0.261; see **Table 2**). The gender differences were statistically significant after age 11, and increased with age from small to medium (Cohen’s *d_1_* = 0.27, *d_2_* = 0.35, *d_3_* = 0.53).

##### Depressive symptoms

The two-way ANOVA on DS yielded statistically significant main effects of gender (*F*[1,1880] = 30.25, *p* < 0.001, η^2^ = 0.016) and age (*F*[2,1880] = 20.33, *p* < 0.001, η^2^ = 0.021), and the expected significant age × gender interaction (*F*[2,1880] = 3.62, *p* = 0.027, η^2^ = 0.004). To further investigate the interaction, two one-way ANOVAs were performed for males and females separately, with age group as a factor. The analyses revealed statistically significant age differences in males (*F*[2,941] = 8.76, *p* < 0.001, η^2^ = 0.018) and females (*F*[2,939] = 14.37, *p* < 0.001, η^2^ = 0.030). *Post hoc* tests using Scheffé’s method showed that DS increased in both male and female students from age 10–11 to age 12–13 (*p* < 0.001), and then decreased in male students from age 12–13 to age 14–16 (*p* = 0.001) back to the prior level (*p* = 0.671). For female students, it remained stable (*p* = 0.656) at the high initial level (compared to age group 10–11, *p* < 0.001; see **Table 2**). Female students reported higher scores on DS than did male students within age groups 12–13 (*t*[556] = -3.30, *p* = 0.001) and 14–16 (*t*[988] = -7.04, *p* < 0.001)—in other words, not in age group 10–11 (*t*[336] = -0.96, *p* = 0.336; see **Table 1**). The gender differences increased in effect size with age from very small to medium (Cohen’s *d_1_* = 0.12 vs. *d_2_* = 0.21 vs. *d_3_* = 0.44).

In a second step, an analysis of covariance (ANCOVA) was conducted to re-analyze the effects of gender (male vs. female) and age (age 10–11 vs. age 12–13 vs. age 14–15) on DS while controlling for SE level and aCSE. Since SE level and aCSE are assumed to influence DS, we expected (1) the two covariates to be statistically significant, and (2) the age × gender interaction to be reduced or eliminated. The ANCOVA revealed that, when controlling for SE level and aCSE, the statistically significant main effect of age held (*F*[2,1877] = 16.46, *p* < 0.001, η^2^ = 0.017), but the main effect of gender became non-significant (*F*[1,1877] = 0.23, *p* = 0.633, η^2^ = 0.000). Additionally, as expected, the gender × age interaction effect disappeared (*F*[2,1877] = 1.15, *p* = 0.316, η^2^ = 0.001). Furthermore, in line with our suggestions, both SE level and aCSE had statistically significant effects on DS (*F*_SE level_[1,1877] = 857.18, *p* < 0.001, η^2^ = 0.314; *F*_aCSE_[1,1877] = 71.91, *p* < 0.001, η^2^ = 0.037).

#### Discussion

The first objective of Study 1 was to clarify the development of DS, SE level, and aCSE along with their gender differences. The purpose of this first part of Study 1 was to provide a foundation for the following investigation of our main hypothesis. To avoid redundancies, these results are discussed in the first part of the general discussion.

Regarding our second goal, the results were in line with the main hypothesis: namely, that aCSE predicts DS. Both level and contingency of SE not only had a significant effect but also accounted for gender and age differences in DS: after controlling for SE level and contingent SE, the main effect of gender and the age × gender interaction disappeared. Furthermore, both covariates were significant, indicating that aCSE had a unique effect beyond SE level on DS.

However, there were two main limitations of this study. First, because of the cross-sectional design, we cannot infer causality. Further research is needed to confirm the assumed causal relationships. Second, a proper test of the diathesis-stress model should include a measure of contingency congruent stressors. It is plausible to assume that students usually do experience academic stressors, such as bad grades; negative feedback from their teachers, parents, and peers; or failure to master a required task. However, in this first study, the stressors congruent with academic contingency were not assessed. Because of these limitations, a second study was conducted.

## Study 2

### Overview

Study 2 was conducted to extend the findings of Study 1 by examining whether aCSE predicts DS prospectively in a sample of university students. Furthermore, as stated earlier, diathesis-stress models of depression suggest that the diathesis and stressor interact to produce DS. Thus, we expected aCSE to serve as a diathesis for the development of DS in the face of stress. To test our hypothesis, students’ levels of aCSE and baseline DS were assessed at the beginning of a semester. To assess participants’ levels of academic stress during the semester, an “academic hassles” self-report measure was administered. At the end of the semester, students completed the DS measure again.

### Method

#### Participants and Procedure

In Study 2, *N* = 545 (74.5% female) students from a large university located in Central Germany participated at Time 1. Students ranged from 17 to 31 years of age (*M* = 22.01, *SD* = 2.80) and were enrolled in a variety of subjects such as psychology, teaching, dentistry, and sports. The majority of students were in their first semester (54.8%); the remaining students were dispersed across higher semesters. Participants were recruited through announcements during introductory classes and via e-mail during the first 2 weeks of the winter semester, which started in October. They were told that the study concerned SE and well-being and were given a URL address to access the online survey. At the end of the winter semester (in March) participants received an email invitation for the Time 2 online survey. Of the 545 students who completed the survey at Time 1, 160 (74.4% female, *M*_age_ = 22.49, *SD*_age_ = 2.76, range_age_ = 19–31) also completed it at Time 2. Full anonymity was guaranteed by giving each participant a code. The items and questions asked in the questionnaires did not go beyond participants’ usual everyday thoughts, we used no misleading cover story, and no experimental manipulation took place. At both measurements, participants took part in a prize draw.

#### Measures

At Time 1 (pretest), the questionnaire included demographic information (i.e., age, gender, year of enrollment, anticipated major) and measures of SE level, aCSE, and DS. At Time 2 (posttest), participants completed measures of DS and academic stress.

##### Self-esteem level

Self-esteem level was measured with the German version of the 10-item RSES (revised German version by Collani and Herzberg, 2003). Sample items included “*At times I think I am no good at all*” and “*I take a positive attitude toward myself.*” Participants responded by indicating their agreement with each item on a scale ranging from 1 (*strongly disagree*) to 5 (*strongly agree*). Negatively worded items were reverse coded; all subscale items were averaged such that higher scores indicated higher SE. The RSES is sufficiently internally consistent (Cronbach’s α = 0.89).

##### Depressive symptoms

The German version of the Center for Epidemiologic Studies Depression Scale (CES-D; Radloff, 1977; Hautzinger and Bailer, 1992) was used to measure current level of DS. The CES-D is a short self-report measure consisting of 20 items to assess depression-related feelings and behaviors such as depressed mood, feelings of guilt and helplessness, sleep disturbance, or psychomotor retardation during the past week (0 = *rarely or none of the time*; 3 = *most or all of the time*). Following the recoding of reverse-scored items, ratings are summed to produce a total score. The possible range of scores is 0–60, with higher scores indicating greater DS. The CES-D had a high internal consistency at both pretest (Cronbach’s α = 0.91) and posttest (Cronbach’s α = 0.92).

##### Academic contingent self-esteem

The extent to which participants’ SE is based on their self-reported academic competence was assessed using the German version (Schwinger et al., in press) of the academic competence subscale of the Contingencies of Self-Worth Scale (CSWS; Crocker et al., 2003b). The academic competence subscale comprises five items, including: “*My self-esteem is influenced by my academic performance*” and “*I feel better about myself when I know I’m doing well academically*.” Responses ranged from 1 (*strongly disagree*) to 5 (*strongly agree*). Following the recoding of reverse-scored items, all subscale items were averaged to produce a total score, with higher total scores indicating higher aCSE. The subscale was sufficiently internally consistent (Cronbach’s α = 0.82).

##### Academic hassles (aHAS)

In the present study, five items were designed to assess self-reported academic hassles (aHAS). Examples of items are “*I have received bad grades*” and “*I had problems mastering my academic exercises*.” Students rated each hassle according to its occurrence over the past week on a dichotomous scale (0 = “*did not occur*” or 1 = “*occurred*”). All items of this scale were summed to create a total score, which ranged from 0 to 5; higher scores reflect greater aHAS. The internal consistency was satisfactory (Cronbach’s α = 0.60).

### Results and Discussion

#### Descriptive Statistics and Intercorrelations

Before testing our main hypothesis, we conducted some preliminary analyses examining the means, standard deviations, and intercorrelations for SE level, aCSE, aHAS, and DS for the entire group and by gender at both measurement times.

The descriptive statistics based on the entire group (final sample, *N* = 160) and by gender and measurement time are presented in **Table 3**. Students had high levels of SE and aCSE. This is consistent with past research findings (e.g., Park et al., 2007). Mean values of aHAS were relatively low in this sample. ANOVA results showed that gender had a statistically significant effect on SE level (*F*[1,158] = 7.21, *p* = 0.007), with men scoring higher than women. Furthermore, ANOVAs revealed a statistically significant effect of gender on aCSE (*F*[1,158] = 5.08, *p* = 0.025), indicating that women had higher levels of aCSE compared to men. No gender differences were found for aHAS (*F*[1,158] = 0.73, *p* = 0.395).

**Table 3:** Mean scores and standard deviations of self-esteem level, aCSE, academic hassles, and DS for the total group and by gender.

	Total	Male	Female
	*M* (*SD*)	*M* (*SD*)	*M* (*SD*)
Self-esteem level	3.94 (0.70)	4.07 (0.59)	3.89 (0.73)
Academic contingent self-esteem	3.65 (0.75)	3.52 (0.84)	3.69 (0.72)
Academic hassles	1.38 (1.34)	1.23 (1.32)	1.44 (1.34)
Depressive symptoms (Time 1)	15.28 (10.27)	12.39 (7.66)	16.27 (10.85)
Depressive symptoms (Time 2)	15.17 (10.56)	14.14 (9.27)	15.55 (11.01)

*N = 160.*

Low levels of DS were found at both times. When using the recommended cut-off scores of >23 on the CES-D (Hautzinger and Bailer, 1992), 18.1% of participants would be classified as “severely depressed” at Time 1 and 20% at Time 2. ANOVA results revealed a statistically significant main effect of gender on DS at Time 1 (*F*[1,158] = 15.16, *p* = 0.000), indicating that females had higher levels of DS. Additionally, we conducted a 2 × 2 (Time × Gender) repeated measures ANOVA on DS. Neither the main effect of time (*F*[1,158] = 0.50, *p* = 0.479) nor that of gender (*F*[1,158] = 3.38, *p* = 0.068) was significant; furthermore, the interaction effect (*F*[1,158] = 2.32, *p* = 0.130) was non-significant.

The correlations of all variables are reported in **Table 4**. aCSE and aHAS were both significantly positively correlated with DS concurrently and longitudinally. Furthermore, SE level showed negative correlations with all other variables. aCSE and aHAS were uncorrelated.

**Table 4:** Correlations between SE level, aCSE, academic hassles, and DS.

	1	2	3	4	5
(1) Self-esteem level	—				
(2) Academic contingency of self-worth	-0.35^∗∗^	—			
(3) Academic hassles	-0.27^∗∗^	0.13	—		
(4) Depressive symptoms (Time 1)	-0.70^∗∗^	0.30^∗∗^	0.29^∗∗^	—	
(5) Depressive symptoms (Time 2)	-0.41^∗∗^	0.12	0.30^∗∗^	0.42^∗∗^	—

*^∗∗^*p* < 0.001.*

#### Primary Analyses

The main goal of Study 2 was to find evidence that aCSE serves as a diathesis for the development of DS in the face of stress. Therefore, we analyzed the data using hierarchical multiple regression to determine whether aCSE interacts with aHAS in predicting changes in DS from Times 1–2. In this regression analysis, we entered gender, SE level, and DS (measured at Time 1) as control variables in Step 1. Then, in Step 2, we included the main effects of aCSE and aHAS. In the final step, Step 3, we added the interaction between aCSE and aHAS. Predictor variables were standardized before creating the interaction term. **Table 5** presents a summary of the regression findings.

**Table 5:** Statistics from regression analyses predicting DS at Time 2.

Variable	*B*	*SE B*	β	*p*
**Step 1**				
Gender	0.68	1.73	0.03	0.69
SE level	-3.11	1.37	-0.23	0.03
DS at Time 1	0.25	0.09	0.27	0.01
**Step 2**				
Gender	0.69	1.70	0.03	0.69
SE level	-2.94	1.38	-0.21	0.04
DS at Time 1	0.23	0.09	0.24	0.02
aCSE	-0.53	0.80	-0.05	0.51
aHAS	1.91	0.78	0.18	0.02
**Step 3**				
Gender	0.68	1.69	0.03	0.69
SE level	-2.55	1.38	-0.19	0.07
DS at Time 1	0.23	0.09	0.25	0.01
aCSE	-0.63	0.79	-0.06	0.43
aHAS	1.53	0.79	0.15	0.05
aCSE × aHAS	1.61	0.78	0.15	0.04

*R^2^ = 0.21 for Step 1; Δ*R*^2^ = 0.03 for Step 2 (*ps* < 0.05); Δ*R*^2^ = 0.02 for Step 3 (*ps* < 0.05).*

**FIGURE 1:**
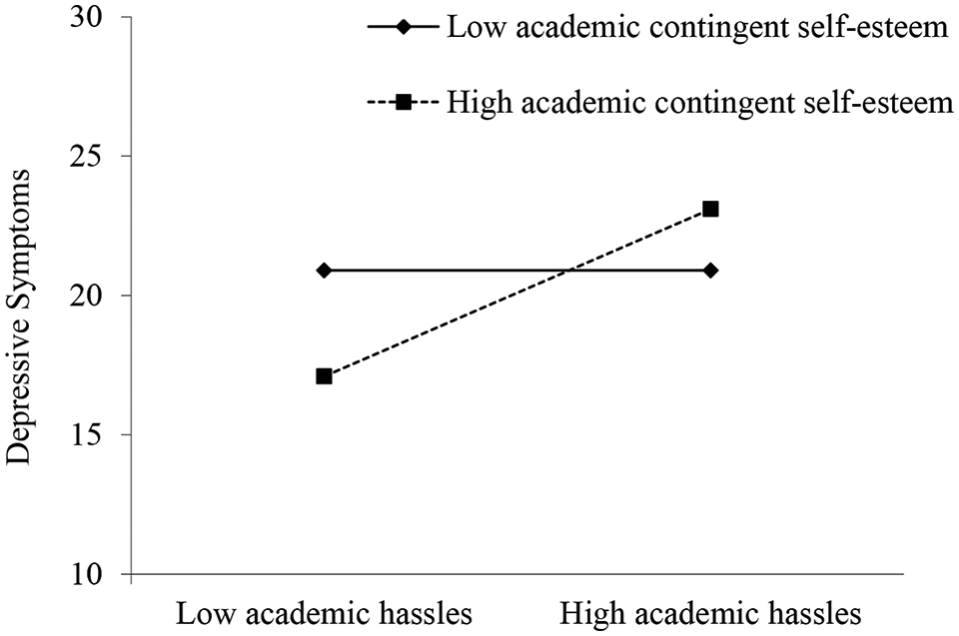
**Study 2: depressive symptoms as a function of aCSE and academic hassles.** Means are plotted at 1 SD above and 1 SD below the mean of aCSE.

At Step 1, DS at Time 1 was a statistically significant predictor of DS at Time 2 (β = 0.27, *p* = 0.007). Furthermore, SE level significantly predicted DS at Time 2 (β = -0.23, *p* = 0.025). No effects were found for gender (β = 0.03, *p* = 0.693). At Step 2, aHAS significantly predicted DS at Time 2 (β = 0.18, *p* = 0.015), while aCSE did not (β = -0.05, *p* = 0.506). Furthermore, the effect of SE level remained statistically significant (β = -0.21, *p* = 0.036). At Step 3, the aSCE × aHAS interaction significantly predicted DS at Time 2 while controlling for DS at Time 1 (β = 0.15, *p* = 0.040). To interpret this interaction, we tested for differences in the effect of aHAS on DS between participants with high and low aCSE (±1 standard deviation from the mean). **Figure 1** shows DS at Time 2—controlled for DS at Time 1—as a function of aHAS and aCSE. As hypothesized, the relation between aHAS and DS at Time 2 was stronger for subjects with high aCSE (*t*[153] = 3.24, *p* = 0.002) than for those with low aCSE (*t*[153] = 0.05, *p* = 0.958). Moreover, when the interaction effect of aHAS and aCSE was added to the final model, the direct effect of SE level on DS at Time 2 was reduced to non-significance (β = -0.19, *p* = 0.067)^1^.

In line with the postulated diathesis-stress model, the reported results showed that people who base their SE on their academic competence and performance are at risk for DS following aHAS. Furthermore, the interaction effects of aCSE and aHAS held when controlling for SE level. Thus, the results demonstrate the incremental value of aCSE in predicting DS.

## General Discussion

In line with models and research viewing SE as a multifaceted construct, the present article focused on contingent SE as a risk factor (over and above that of SE level) for the development of DS. While we consider contingent SE vulnerability across all age groups and several domains, in the present studies, we focused on adolescence (a life stage when DS increases and gender differences emerge) and the academic domain of contingent SE. In two studies, we attempted to investigate the relationship between aCSE and self-reported DS in adolescence and young adulthood.

Another goal of our studies was to examine the unique predictive values of both aCSE and SE level. Previous studies have already linked contingent SE to depression among college students (Sargent et al., 2006) and adolescents (Burwell and Shirk, 2006). However, these studies yielded mixed results, and did not investigate the incremental effects of contingent SE beyond SE level. Overall, our results supported the main hypothesis—namely, that contingent SE is a psychological risk factor over and above SE level for the development of DS. Adolescents whose SE is derived from their academic competence and achievements (i.e., those with high aCSE) may be at higher risk for developing DS compared to adolescents with lower aCSE.

The first purpose of Study 1— which preceded investigation of our main research question—was to explore aCSE, SE level, and self-reported DS among adolescents and describe age and gender differences in these variables to provide background for further analyses. The overall trend for adolescents from age 10–11 to age 14–16 could be roughly described as follows: as expected, there were no significant gender differences before puberty. However, after puberty, gender differences emerged and increased up to a medium effect size in students aged 14–16 years. Compared to boys, girls showed greater DS, lower SE level, and greater aCSE. While the direction of gender differences was in line with our expectations, the differences were rather small. Additionally, the observed trends were inferred only from cross-sectional data. Therefore, the development of these variables must be investigated in a longitudinal design. Future research should also look into the observation that after the onset of puberty, boys (but not girls) seem to return to their more adaptive combination of higher SE level and lower aCSE (and lower DS).

Regarding the second aim of Study 1, the results supported our assumption that gender and age differences in depression, specifically the increase in DS in girls after the onset of puberty, are in part due to aCSE. After controlling for aCSE (and SE level), both the significant age × gender interaction and the gender main effect on DS disappeared (the main effect of age, however, remained, suggesting that variables other than SE level and aCSE contributed to the increase). This pattern, together with the finding that aCSE predicted the amount of self-reported DS over and above SE level, provided not only preliminary support for our main hypothesis of the causal role of contingent SE in the development of DS, but also highlights the need to study SE in all its facets when explaining the onset of depression.

Following our theoretical considerations, aCSE is supposed to interact with academic stressors (e.g., verbal or non-verbal feedback, struggling with tasks, upward social comparisons) in the development of DS. A limitation of Study 1 is that these negative events were assumed to be a part of everyday life at school, but were not actually assessed. Moreover, the cross-sectional design of Study 1 did not provide evidence for the postulated causal relation. Thus, while the results of Study 1 are in line with our main assumption that aCSE is one cause of the increasing rates in DS in puberty, the limitations of the first study required us to conduct another second study to confirm the causality and test the underlying diathesis-stress model more adequately.

The results of Study 2, conducted in a sample of late adolescents and young adults, show that aCSE interacts with academic stress to predict change in self-reported DS over the course of 6 months. More precisely, students whose SE is dependent on academic competence and achievements were more vulnerable to developing DS when they experienced high levels of academic stress. Whereas previous studies failed to show a significant interaction between aCSE and academic stress in predicting changes in DS (Burwell and Shirk, 2006) our findings are in line with the assumption of the diathesis-stress model of depression—namely, that the combination of a diathesis and a stressor leads to depression. Furthermore, the interaction effects of aCSE and academic stress held when controlling for SE level. Thus, our results provide support for the incremental contribution of aCSE to DS. Moreover, our results support the notion that contingent SE in concert with self-relevant stressors is a more important vulnerability marker for depression than is SE level. Specifically, additional analyses revealed no evidence for an interaction effect between SE level and academic stress in predicting DS. The results indicate that contingent SE is an important vulnerability factor for DS, whereas decreased SE level seems to be a symptom of depression. Therefore, future research investigating the role of SE in depression should consider contingent SE.

### Limitations

The main limitations of Study 1 lie in the research design, which does not allow for causal conclusions or testing of the postulated diathesis-stress model. These limitations were compensated by Study 2, wherein we used a longitudinal design and assessed how stressors interact with contingent SE in the prediction of DS. However, a limitation of Study 2 is that we used university student samples—namely, late adolescents and young adults—to confirm the causal role of aCSE in DS. Overall, future research will have to use a prospective design with children and young adolescent samples to investigate whether increases in aCSE during puberty paired with increases in academic stress are predictive of DS as well as diagnosed major depression disorder in late adolescence and adulthood.

In both studies, SE level, aCSE, and DS were assessed using self-report questionnaires, which can be subject to bias. Unfortunately, there are no valid objective methods of assessing SE level or aCSE. Indeed, implicit measures do not seem to target the same construct, and we believe that inferring contingent SE from behavior is premature at this stage. Regarding DS, the assessment could probably be improved by including data from different measurement methods, such as behavioral data, physiological measures, or others’ reports.

### General Implications for Future Research

Addressing the question of the mechanism by which aCSE results in DS following academic stress was beyond the scope of this paper. However, the investigation of potential mediating factors is fruitful in understanding how contingent SE leads to depression. Some researchers suggest that instability of SE functions as a mediator of the effect of contingent SE and congruent stressors on DS (Crocker and Wolfe, 2001; Lopez et al., 2014). According to these theorists, negative events congruent with the contingent SE cause instability in SE, which in turn leads to depression. Lopez et al. (2014) were the first to yield supporting evidence for this assumption. Additionally, Cambron and Acitelli (2010) provided a further process model of depression, in which friendship contingent SE serves as a vulnerability factor for the development of depression following negative friendship events through dysfunctional cognitive and behavioral patterns (e.g., excessive reassurance seeking, negative feedback seeking, rumination). Based on these results, it might be promising to investigate whether the same mechanisms mediate the effect of aCSE and academic stress on depression.

In the present paper, we focused on aCSE as a risk factor for depression. It should be mentioned that any external SE contingency is assumed to generate DS following negative life-events congruent with that contingency. Specifically, in adolescence, physical appearance becomes an even more important domain of self-worth, particularly among girls (Burwell and Shirk, 2006, 2009). At the beginning of puberty, girls are confronted with tremendous body changes, such as changes in the body-fat-to-muscle ratio. How easily they deal with those body changes depends most of all on how closely their physical appearance matches their female body ideals, which are influenced to a large extent by media images (Burwell and Shirk, 2009). These well-defined stereotypes of perfect bodies and faces are almost unattainable for ordinary girls. Internalization of these ideals inevitably leads to negative real vs. ideal self-comparisons, which result in body dissatisfaction, maladaptive self-validation (e.g., dieting), and negative affect, especially among girls whose SE is highly contingent on their physical appearance. Thus, girls with high levels of appearance contingent SE are confronted with numerous threats/attacks to their SE and are at heightened risk for the development of DS. Future research should test the generalizability of the postulated diathesis-stress model across different domains of contingencies of SE, in particular in the domain of appearance.

### Practical Implications

The present study has important implications for prevention and intervention in the field of SE and depression. Previous prevention and intervention programs have primarily focused on the level of SE. However, studies have shown that strategies intended to boost SE level, such as positive self-statements and success induction, can be useless or even backfire. Especially among low SE individuals, positive self-statements and experiencing success on intellectual tasks can trigger negative self-relevant thoughts, anxiety, and physical symptoms (Wood et al., 2006). A possible explanation for these harmful effects is that these SE interventions do not change individuals’ contingent SE. For individuals whose SE is highly contingent on academic competence or achievement, success may focus attention to unfulfilled self-standards or lead to even higher standards that individuals anticipate or fear falling short of achieving (cf. Wood et al., 2006). Thus, as long as dysfunctional contingent SE persists, these methods and programs are ineffective or even contraindicated. The results of the present studies indicated that prevention and intervention programs should also change the extent to which peoples’ SE is contingent upon high self-standards of excellence or minimize the impact of contingent SE on depression instead of trying to change SE level. Intervention research that focuses on how to change contingent SE is rather rare, and thus cannot currently be used to influence practical application. Nonetheless, some researchers have suggested ways of reducing contingent SE or reducing its costs (Crocker and Park, 2012).

One possibility for reducing the costs of maladaptive contingent SE is self-compassion. Derived from Buddhist beliefs, self-compassion contains three basic aspects: self-kindness, common humanity, and mindfulness. Self-compassion involves a positive view of the self: self-compassionate people behave in a kinder and gentler way toward themselves because they recognize that failure is an inevitable part of being human. Specifically, personal failings and imperfection are parts of the shared human experience. Therefore, failure does not separate us from others, but rather connects us with them. Thus, personal inadequacies can be accepted and acknowledged with self-kindness. Contrary to contingent SE, self-compassion might be an adequate alternative for dealing with negative life events that buffer people against threats to SE. In support of this idea, studies have shown that self-compassion is negatively correlated with contingencies of SE, particularly with external contingencies such as social approval, appearance, and performance (Neff and Vonk, 2009). Self-compassion interventions have shown significant effects on various variables, including increased self-compassion, mindfulness, optimism, and self-efficacy and decreased rumination (Smeets et al., 2014).

In order to develop early preventive programs for children and adolescents, we must expand our knowledge about the developmental characteristics of contingent SE. Developmentally, different aspects of socialization such as bonding experiences are hypothesized to contribute to contingent SE. Park et al. (2004) investigated the relation between contingent SE and attachment styles. The results suggest that individuals with insecure attachment styles—namely, those who had inconsistent (punitive and benevolent) parents—doubted whether they were worthy of the love of their family. Because family support is not a trustful or available source of SE for these individuals, they must validate their worth and value as a person through different avenues, such as by looking good or being physically attractive (Park et al., 2004).

Although early interaction with caregivers plays an important role in influencing individuals’ contingent SE, it is likely that later relationships with significant others are another important basis of socialization for children and adolescents and thus for developing contingent rather than non-contingent SE. Even though contingent SE has its roots in early childhood, teachers, parents, and peers might nurture existing contingencies. The association between success or failure and “being a valuable person” should not be reinforced. Instead, it would be beneficial that teachers and parents direct their feedback to the process of learning. Creating lessons with learning goals might reduce the threat to SE and hence the costs of aCSE (Niiya et al., 2004). In this vein, O’Keefe et al. (2013) have shown that creating a mastery-goal-structured educational context temporarily reduces students’ contingent SE. Communicating that “*making a mistake is not being a mistake*” as well as focusing on learning and improvement instead of promoting self-validation goals might be an adequate approach to reduce contingent SE, and subsequently, DS. Even though contingent SE is a powerful source of motivation, its costs seem to outweigh the benefits (Crocker and Park, 2004). As discussed in this paper, an elevated risk of developing DS during adolescence is one of the costs of contingent SE.

## Conflict of Interest Statement

The authors declare that the research was conducted in the absence of any commercial or financial relationships that could be construed as a potential conflict of interest.

## Footnotes

1 An additional hierarchical regression analysis was conducted to examine, whether SE interacts with aHAS in predicting change in DS from time 1 to time 2. In Step 1, we controlled gender and DS at time 1. In Step 2, we entered SE and aHAS into the regression. In Step 3, we added the product term of SE and aHAS (both z-standardized) to the regression equation. Analysis results revealed a significant effect for aHAS (β = 0.15, *p* < 0.05). Neither the effect of SE (β = -0.08, *p* > 0.05), nor the interaction effect (β = -0.18, *p* > 0.05) was significant in predicting time 2 DS – controlling time 2 DS.
